# Effect of IgY antibody coated fish feed in preventing motile *Aeromonas* septicaemia in *Ctenopharyngodon idella*: a histopathological perspective

**DOI:** 10.3389/fimmu.2026.1885532

**Published:** 2026-08-18

**Authors:** Thushara James, Mujeeb Rahiman K.M., Denoj Sebastian, Suja Rani S, Dinesh M.D, Pratheesh Prakasam Thanka

**Affiliations:** 1 Department of Aquaculture and Fishery Microbiology, MES Ponnani College, Ponnani, Kerala, India; 2 School of Industrial Fisheries, Cochin University of Science and Technology, Cochin, Kerala, India; 3 Department of Life Sciences, University of Calicut, Malappuram, Kerala, India; 4 Department of Veterinary Pharmacology and Toxicology, College of Veterinary and Animal Sciences, Mannuthy, Thrissur, Kerala, India; 5 Department of Microbiology, Nehru Arts and Science College, Coimbatore, Tamil Nadu, India; 6 National Water and Energy Centre, United Arab Emirates University, Abu Dhabi, United Arab Emirates

**Keywords:** *Aeromonas hydrophila*, aquaculture immunotherapy, *Ctenopharyngodon idella*, IgY, MAS

## Abstract

*Aeromonas hydrophila* is an opportunistic bacterial pathogen responsible for motile *Aeromonas* septicaemia (MAS), a major disease affecting freshwater aquaculture species such as *Ctenopharyngodon idella* (Grass carp). The conventional use of antibiotics to control bacterial infections has led to several adverse outcomes, including antibiotic resistance, drug residues in fish tissues, and environmental pollution. The present study aimed to develop and evaluate the efficacy of immunoglobulin Y (IgY) antibodies as an alternative therapeutic and preventive measure against *A. hydrophila* infections. The median lethal concentration (LC_50_) of *A. hydrophila*, determined through immersion challenge, was 1.5 × 10^7^ CFU/mL. Fish feed coated with 3.5–5% IgY was administered to experimental groups, resulting in survival rates ranging from 65% to 100%. The therapeutic, neutralization, and prophylactic efficacies of IgY treatment were observed to be 60%, 70%, and 80%, respectively. Histopathological examinations revealed that IgY-treated fish showed minimal pathological alterations in the skin, gills, small intestine, and large intestine compared to live bacterial challengedand control groups. The results demonstrate that oral administration of IgY-coated feed effectively reduces *A. hydrophila* infections and related tissue damage. This study highlights IgY as a promising, environmentally friendly immunotherapeutic alternative to antibiotics in aquaculture, offering a sustainable approach for disease prevention and management in *C. idella* culture systems.

## 1 Introduction

Aquatic organisms have historically played a crucial role in global human nutrition, providing substantial amounts of dietary protein and micronutrients, and they significantly contribute to nearly all of the United Nations Sustainable Development Goals (SDGs) for Agenda 2030 (1, 2). The global fisheries and aquaculture production in 2022 surged to 223.2 million tonnes, a 4.4 per cent increase from 2020, according to the 2024 edition of The State of World Fisheries and Aquaculture (SOFIA). Aquaculture now surpasses capture fisheries as the primary source of aquatic foods worldwide. For instance, the global aquaculture market was valued at around USD 244 billion in 2023 and is expected to grow at a compound annual growth rate (CAGR) of 4–5% over the next decade (3). This expansion is driven by increasing demand for high-quality protein, expanding middle-class consumption, and the intensification of production systems in both freshwater and brackish water environments. Freshwater aquaculture, in particular, has experienced significant growth due to more accessible infrastructure, abundant inland water resources, and a diverse range of species such as carps, tilapia, and freshwater crustaceans (4).

However, the sector faces multiple constraints: limited availability of high-quality seed, competition for land and water, inadequate access to feed and technology at the grassroots level, and increasingly frequent environmental challenges (e.g., climate-driven disasters). These constraints, when combined with disease outbreaks and high mortality rates, can severely undermine fish farmers’ livelihoods, threaten food security, and weaken the economic sustainability of aquaculture in developing regions (5, 6).

Among disease-causing agents in aquaculture, *A. hydrophila* is a well-known opportunistic pathogen responsible for a broad spectrum of infections, including ulcer disease, red-sore disease, haemorrhagic septicaemia, and MAS in both freshwater and brackish water fishes (7, 8). Virulence factors such as hemolysin (hlyA), aerolysin (aerA), cytotoxic heat-labile enterotoxin (act), and cytotonic heat-labile and heat-stable enterotoxins (alt, ast) contribute to pathogenicity and systemic disease in immunocompromised fish (9). The widespread prophylactic and therapeutic use of antibiotics in aquaculture has accelerated the emergence of antimicrobial-resistant aquatic bacteria, thereby limiting treatment options and raising environmental concerns (10, 11). Contemporary reviews emphasise that antibiotic resistance in aquaculture pathogens not only diminishes the efficacy of antimicrobials but also increases the risk of resistance gene transmission into native aquatic microbial communities (12).

Given these challenges, the industry increasingly seeks alternative, sustainable, environmentally friendly, and effective disease-control strategies. One promising approach is the use of avian egg-yolk-derived antibodies, immunoglobulin Y (IgY), as a form of passive immunotherapy. IgY antibodies, naturally found in birds, reptiles, and amphibians and considered the evolutionary precursors of mammalian IgG and IgE can be extracted non-invasively from egg yolks (13). They have been explored in prophylactic and therapeutic contexts for both terrestrial and aquatic animals (14). Recent reviews highlight their potential in aquaculture disease management, demonstrating efficacy in preventing bacterial and viral infections when administered *via* feed, encapsulation, or immersion (15, 16). Beyond its primary role in bacterial neutralization, IgY has been shown to exert significant immunomodulatory effects, effectively recalibrating the host’s immune-axis and restoring gut microbial homeostasis (17). While the potential of IgY as an antibiotic alternative has been recognized in aquaculture, this study significantly advances the field by addressing the critical gap between laboratory-scale antibody production and practical, field-stable delivery systems for *C. idella*. Furthermore, this study provides efficacy of IgY-coated fish feed by passive immunization to restore structural integrity and reduce systemic mortality, offering a standardized protocol for large-scale, antibiotic-free disease management in the grass carp industry.

## 2 Materials and methods

### 2.1 Materials

In this study, we used 22 to 24-week-old white leghorn chickens as experimental animals to produce IgY. Additionally, we employed two-month-old *C. idella* as subjects to investigate antigen-antibody reactions. Egg-laying chickens were purchased from a small animal breeding station at the College of Veterinary and Animal Sciences, Mannuthy, Thrissur. The experimental fish, weighing 12-15g and measuring 8 ± 2cm in length, were obtained from Ponnani, Malappuram, Kerala, and housed 10 per aquarium with 1 cubic foot of space. Strains of *A. hydrophila* (Microbial Type Culture Collection and Gene Bank – 1739) were obtained from the Council of Scientific and Industrial Research- Institute of Microbial Technology, Chandigarh, India. For this experiment, we adhered to the Prevention of Cruelty to Animals (PCA) Act 1960, Breeding and Experimentation Rules 1998, and other amendments issued by the Government of India. All these experiments were reviewed and approved by the Institutional Bio-Safety Committee and the Institutional Animal Ethics Committee at Mannuthy, Kerala, India (IBSC Approval No: IBSC-21-13, IAEC Approval No: IAEC/CVASMTY16/2021).

### 2.2 Determination of LC_50_

The experiments were conducted in dechlorinated, well-aerated water under a constant photoperiod (12 h light: 12 h dark). The water conditions were maintained twice in a week as follows: temperature 28 ± 2°C, pH 7.4 ± 0.2, dissolved oxygen (DO) ≥ 6.5 mg/L, and total ammonia nitrogen < 0.01 mg/L. To determine the median LC_50_, *A. hydrophila* was cultured and the bacterial load was adjusted to concentrations of 5**×**10^6^, 10**×**10^6^, 15**×**10^6^, 20**×**10^6^, 25**×**10^6^ and 30**×**10^6^ CFU/mL. These varying concentrations were then used to treat fish in the study, where the fish were kept in 1000 mL tanks during this period. The fish were observed for 96 hours following treatment, with 10 fish per tank considered as a single unit. Data were carefully recorded throughout this period and compared with the control group of fish free of pathogens. The concentration at which 50% of the fish population succumbed to bacterial infection was identified as the LC_50_ value (18). This value is a crucial parameter in understanding the virulence of *A. hydrophila* and its impact on fish populations.

### 2.3 Production and purification of IgY antibodies

Formaldehyde-killed cells of *A. hydrophila* were injected intramuscularly at multiple sites in the breast muscles of chickens. The chickens received subsequent booster injections (Mac Farland’s standard – 0.5 to 8) at 7-day intervals *via* the same route of administration. Eggs were collected based on the presence of *A. hydrophila* antibodies in the serum, confirmed by test bleeding. The antibodies were extracted from egg yolk using a dual extraction method with polyethylene glycol and ammonium sulphate (19). Salts and impurities were removed by dialysis, and the IgY was further purified through ion exchange chromatography (20, 21). The purity of chicken egg yolk antibodies was checked by SDS-PAGE (22). The image were analyzed using ImageJ software (National Institutes of Health, Bethesda, MD, USA). The protein content of the eluted fractions was estimated by the Lowry protein assayd using Folin-Ciocalteu phenol reagent as one of its reagents (23). Bovine serum albumin (BSA) was used as the standard protein, and the protein concentration of the samples was calculated from the BSA standard calibration curve.

The antibody titre against *A. hydrophila* was determined by indirect ELISA. Nunc Polysorp ELISA plates were coated with 200 µL/well of a live log-phase *A. hydrophila* bacterial suspension (5 × 10^6^ cells/mL) prepared in 0.05 M carbonate-bicarbonate coating buffer (pH 9.6). Purified egg yolk IgY served as the primary antibody and was tested at serial dilutions of 1:10, 1:100, 1:1,000, 1:10,000, and 1:100,000 (100 µL/well). The assay was completed according to the standard indirect ELISA protocol described by Voller et al. (1976) (24). The antibody titre was defined as the highest IgY dilution that produced a measurable absorbance above the negative controls (PBST and non-specific IgY). The *A. hydrophila* sample was tested separately in triplicates.

### 2.4 Development of antibody-coated fish feed

To ensure the uniform distribution of IgY across the feed matrix, 10g feed samples were placed in a rotary mixer. The specific IgY solutions (2.5% to 5%) were prepared in sterile saline. The 24 mL solutions were applied to the feed using a slow-drip method during continuous mechanical agitation for 15 minutes, ensuring homogenous absorption rather than simple surface coating. Following saturation, the feed was spread in a single layer on sterile trays and subjected to low-temperature air-drying (25–30°C) for 12 hours. This prevented the thermal denaturation of the IgY protein. The uniformity of the coating was verified by randomly sampling pellets from different sections of the tray and measuring total protein content, which confirmed a variance of less than 5% across the samples. We also assessed the impact of different IgY concentrations in feed on fish exposed to the LC_50_ pathogen concentration. The aim was to determine the minimum IgY dose in the feed required to protect more than 50% of the fish from mortality (10 fish in each tank).

### 2.5 Experimental setup for *in-vivo* studies

After acclimatisation and observation for a week, the fish were transferred into the experimental tank and categorised into three groups (**Table 1**). Where, the fish were fed once daily at 5% of their total body biomass throughout the experimental period. The impact of varying IgY concentrations with different modes of application was examined. Group 1 served as a control and was kept free from bacterial pathogen. Group 2 studied the infectivity and rate of mortality caused by the bacterial culture by adding live bacterial suspension. In the survival study (Group 3), fish were treated in three different phases. In phase 1, fish were treated with live bacterial challenge and antibody-coated fish feed simultaneously to assess *in-vivo* neutralization efficacy. In phase 2, fish were first treated with live bacterial challenge for 3 days, then fed antibody-coated fish feed until the 14^th^ day to evaluate the therapeutic potential of IgY. In phase 3, fish were exposed to IgY antibodies through coated fish feed for 7 days, then challenged with live bacterial pathogen up to the 14^th^ day to examine the prophylactic potential of IgY. After completion of the immersion challenge, the bacterial suspension was discarded, and the partial water replacement were carried out to maintain optimal water quality throughout the study. Three experimental replicates was maintained in a separate tank throughout the study. Challenge water was not shared between replicates, thereby preventing cross-contamination and ensuring independent experimental conditions. The fish were observed throughout for their health status, behaviour, and any signs of disease.

**Table 1:** Grouping of experimental animals for *in-vivo* studies.

Group		Mode of application	Fish treated as
Control		Nil	Fish without exposure to bacterial pathogens
Infectivity study		Immersion method	Fish exposed to live culture of bacterial pathogens^#^
Survival study	Phase 1 (Neutralization)	Immersion method and feeding method	Fish was treated with live bacterial challenge and simultaneously fed with antibody-coated fish feed*
	Phase 2 (Curing/therapeutic)	Immersion method and feeding method	Fish was treated with live bacterial challenge for 3 days, then provided antibody-coated fish feed up to14^th^ day*
	Phase 3 (vaccination/prophylactic)	Immersion method and feeding method	Fish fed with antibody-coated fish feed for 7 days and then treated with live bacterial challenge for 7 days*

^#^ 15×10^6^ CFU/mL; *3.5% of antibody/g of feed.

### 2.6 Histopathology analysis

At the completion of the *in vivo* experiments, all surviving fish were humanely euthanized by immersion in a clove oil solution. Pure clove oil containing approximately 90–95% eugenol was prepared as a 1:9 stock solution in 92.8% ethyl alcohol to facilitate dispersion in water. A volume of 0.20 mL of pure clove oil was added to 500 mL of water, corresponding to a final concentration of 0.40 mL/L (400 µL/L), following the method described by Fernandes et al. (2017) (25). Fish were individually maintained in the euthanasia solution until complete cessation of opercular movement and pectoral fin activity and were retained in the solution for an additional 30 min to ensure death. Death was confirmed by the absence of opercular movement and response to external stimulation. Tissues from euthanized fish and fish that died during the experimental infection were immediately collected, fixed in 10% neutral buffered formalin, and processed for histopathological examination. The euthanasia procedure was performed in accordance with the relevant institutional animal ethics and applicable animal welfare guidelines.

### 2.7 Data analysis

The results were analyzed statistically using SPSS (Statistical Package for the Social Sciences) version 28, produced by IBM (International Business Machines) Corporation, Armonk, New York, United States, and the graphs were plotted using OriginPro 8.1, developed by OriginLab Corporation, Northampton, Massachusetts, United States.

## 3 Results

### 3.1 *In-vitro* and *in-vivo* analysis

**Figure 1:**
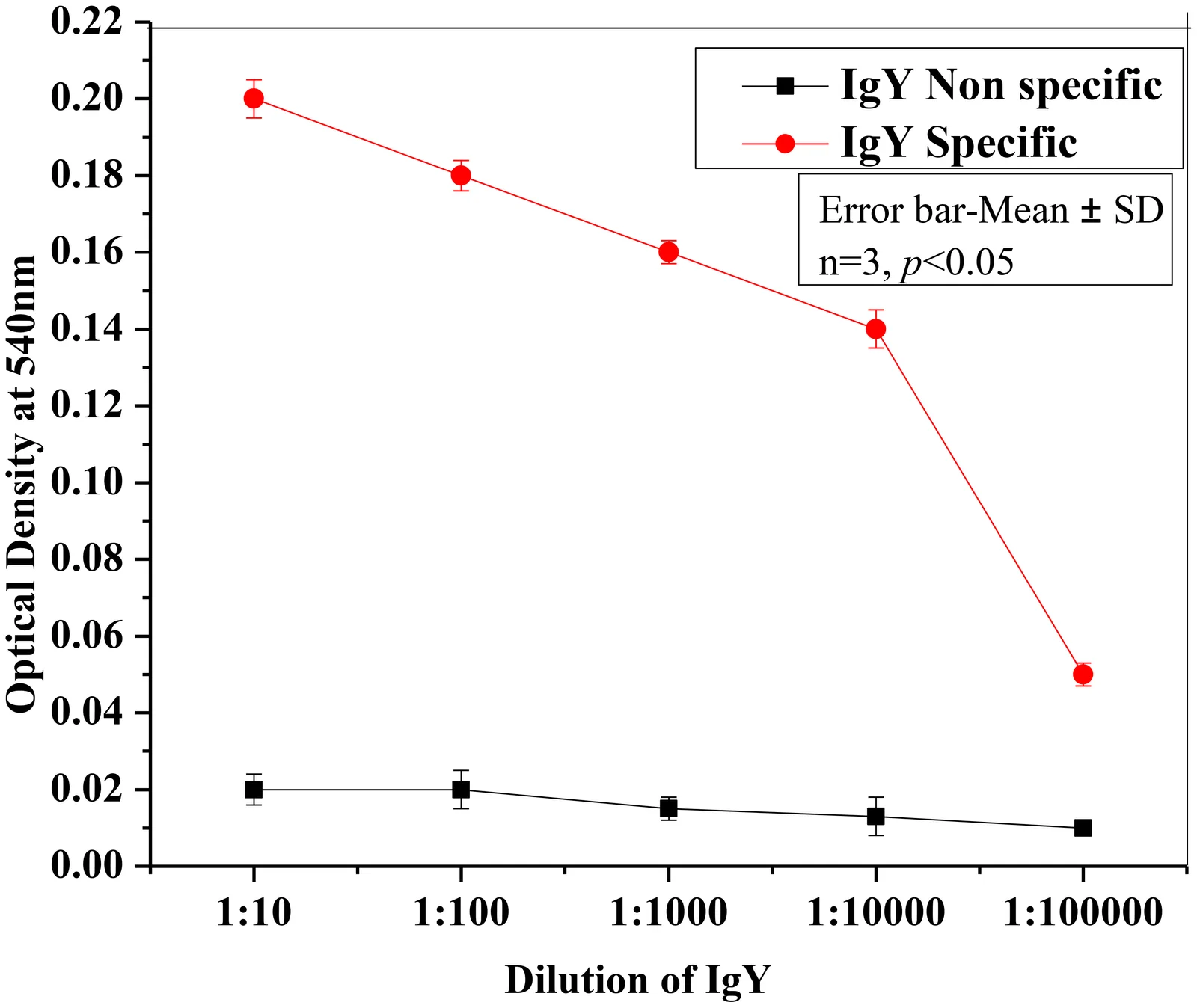
ELISA titration of specific IgY against non-specific IgY.

**Figure 2:**
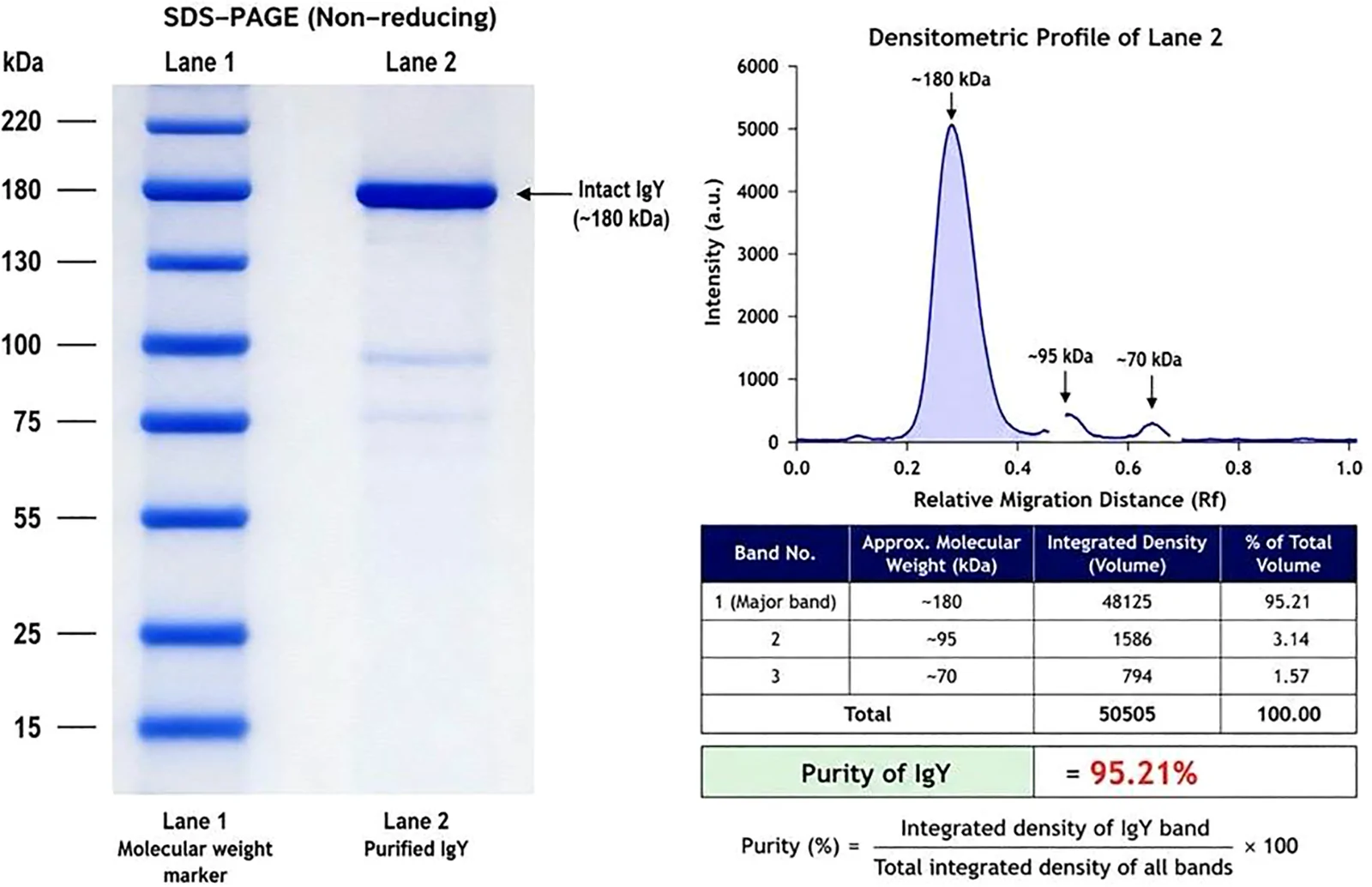
SDS-PAGE analysis of IgY antibodies. Where, Lane 1 is molecular marker band and Lane 2 is IgY and its densitometric profile of lane 2.

**Figure 3:**
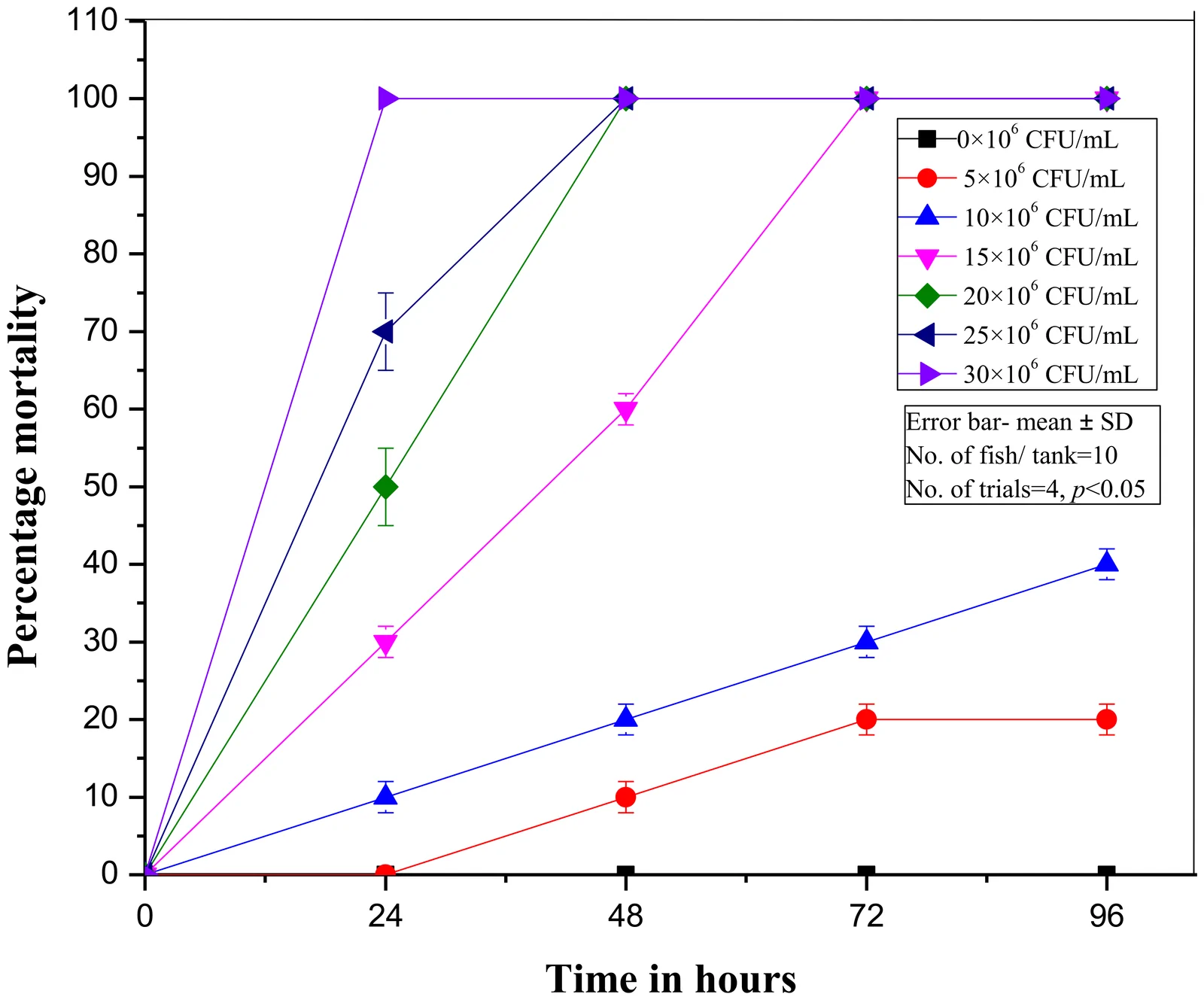
Determination of the LC_50_ of *Aeromonas hydrophila* against *Ctenopharyngodon idella*.

The purified IgY antibodies yielded an average protein content of 11.88 mg/mL, with high titers observed at 1:10,000 dilutions *via* ELISA, confirming strong specificity to *A. hydrophila* antigens (**Figure 1**, Two-way ANOVA followed by Tukey’s multiple comparison test, n=3, p<.05). The 180 kDa IgY antibodies were confirmed *via* SDS-PAGE analysis under non-reducing conditions, showing 95.21% purity. The results were compared with the broad-range molecular marker (Genei, Pvt Limited, Bangalore) (**Figure 2**). These analyses validated the determination of the LC_50_ value of *A. hydrophila* as 15×10^6^ CFU/mL at 48 hours, demonstrating significant mortality rate differences across various bacterial concentrations (**Figure 3**, Welch’s ANOVA, *p* < 0.05).

**Figure 4:**
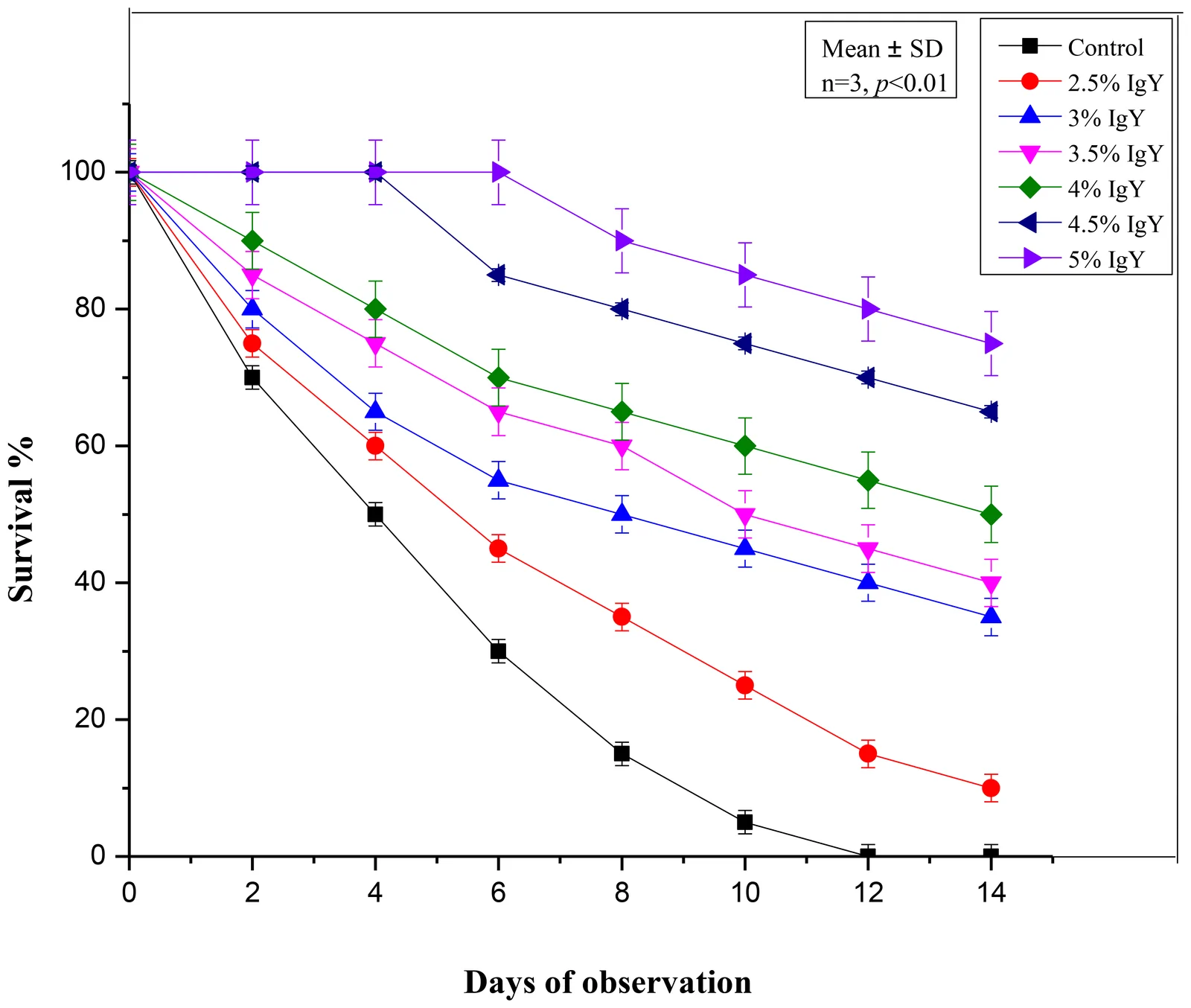
Survival kinetics of *C. idella* challenged with an LC_50_ dose of *A. hydrophila* following treatment with varying concentrations of IgY-coated feed (2.5% to 5%).

Furthermore, survival analysis demonstrated a clear dose-dependent protective effect, with 3.5% IgY-coated feed providing approximately 50% survival by day 10, while higher doses (4.5% and 5%) offered even greater protection (**Figure 4**). Statistical analysis using the Kaplan–Meier method confirmed that differences among treatment groups were highly significant (*p* < 0.01). Additionally, survival proportions at day 10 were compared using the Chi-square test (*p* < 0.001, χ² = 67.64, df = 6), which also showed significant differences among groups.

**Figure 5:**
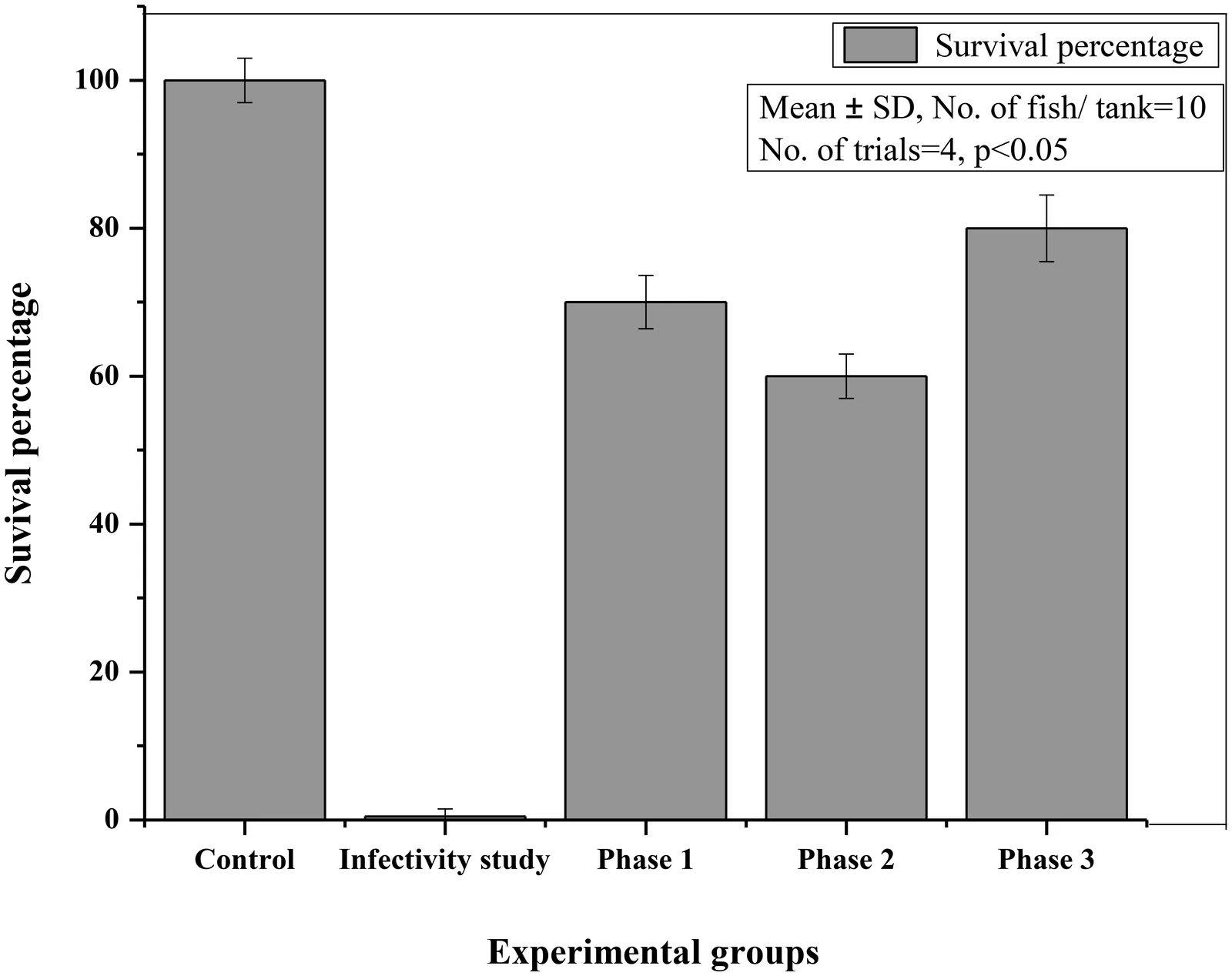
Survival outcomes of *C. idella* for 14 days with *A. hydrophila* under different IgY treatment strategies. Where, Phase 1- neutralization study, Phase 2- therapeutic, and Phase 3- prophylactic.

Phase-1 of the survival study (neutralization study) demonstrated excellent neutralization capacity of IgY antibodies, with a 70% recovery rate. In phase 2 (curing/therapeutic study), the curing ability of IgY antibodies was assessed, resulting in a 60% survival rate after 14 days. In phase 3 (prophylactic study), IgY was employed as a prophylactic agent and achieved an 80% survival rate after 7 days of challenge with *A. hydrophila* pathogen. The Chi-Square test showed significant differences in survival rates among the groups (**Figure 5**, p < 0.05), confirming the efficacy of IgY treatment.

### 3.2 Histopathological analysis of fish specimens

The histopathological analysis of tissue samples from skin, gills, small intestine, and large intestine under control, infected, and IgY-treated conditions revealed significant differences in infection status (Chi-Square, *p* < 0.05). In the control group, all samples remained uninfected, confirming the absence of pathogen exposure. In the infected group, all samples showed infection, confirming the pathogenic effect. The IgY-treated group displayed a substantial number of cured tissue samples across all types, indicating the effectiveness of the IgY antibodies in treating infection.

**Figure 6:**
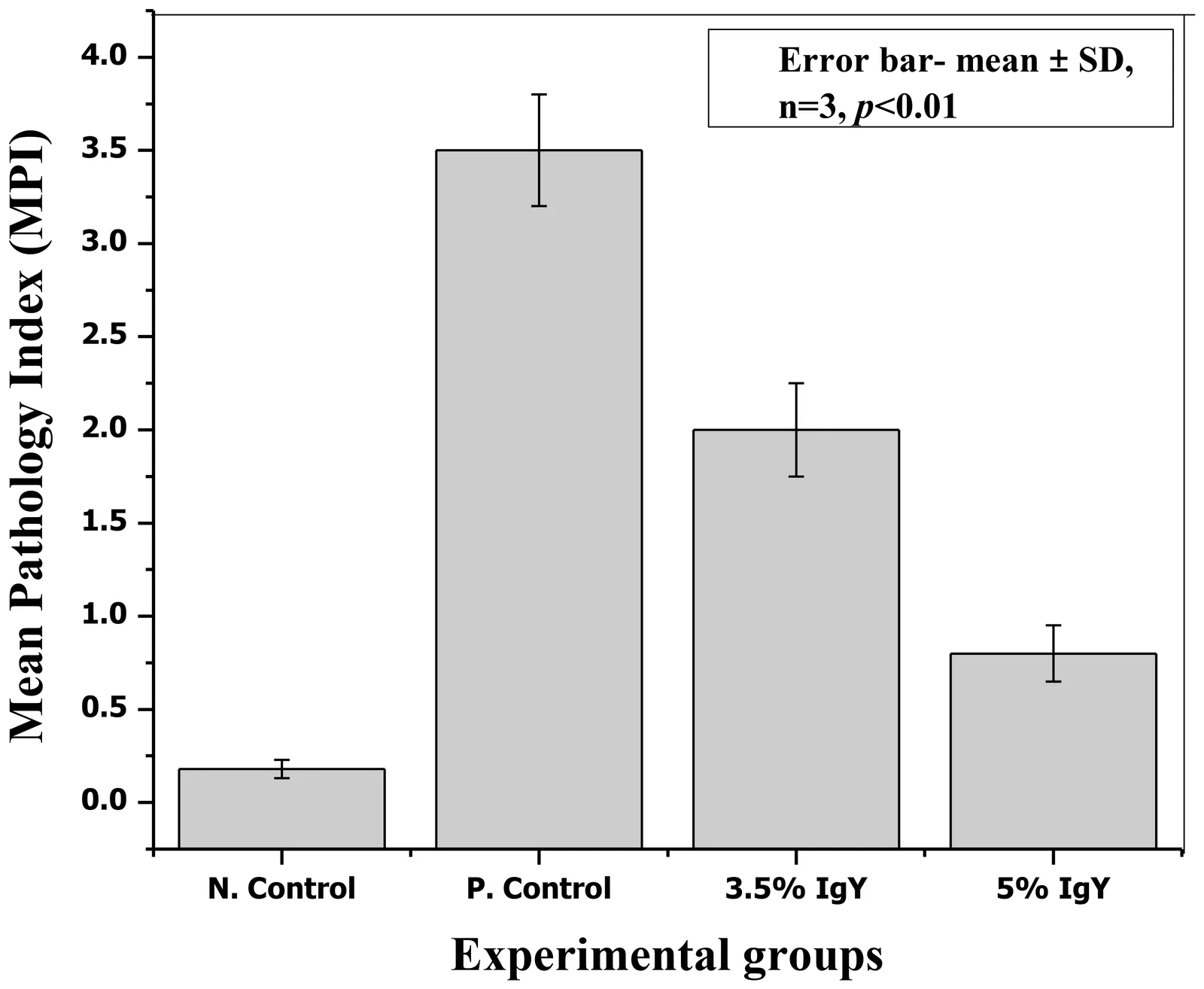
Mean Pathology Index (MPI) of *C. idella* following *A. hydrophila* challenge and treatment with IgY-coated feed. The uninfected fish in this study exhibit continuous, rigid skin features without any signs of infection (**Figure 7a**). However, the histopathology analysis of the infected fish revealed ruptured skin epithelium, oedematous with collagenous and inflammatory infiltrates (**Figures 7b, c**). In contrast, the IgY-treated fish skin showed a structure similar to that of uninfected fish (**Figure 7d**).

The pathogen-free negative control group exhibited clear, baseline structures (MPI < 0.2) with no evidence of pathological deviations across all tissues (**Figure 6**, *p* < 0.01). In stark contrast, the infected positive control group demonstrated severe structural aberrations (MPI > 3.5), characterized by complete lamellar destruction and terminal clubbing in the gills, alongside total necrotic sloughing of the intestinal mucosa. Administration of 3.5% IgY-coated feed provided moderate protection (MPI ≈ 1.0), where tissue damage was limited to partial lamellar fusion in the gills and mild inflammatory cell infiltration within the submucosa of the large intestine. Notably, the 5% IgY-coated feed group showed the highest efficacy, exhibiting low structural ruptures and minimal necrosis (MPI < 1.0); in this group, the intestinal villi remained largely intact with only focal epithelial lifting, and the skin layers displayed no significant lesion formation, closely resembling the healthy control architecture. Bars represent the mean ± standard deviation (SD) from three independent experimental trials (n = 3). Statistical analysis was performed using one-way ANOVA followed by Tukey’s multiple comparison test.

**Figure 7:**
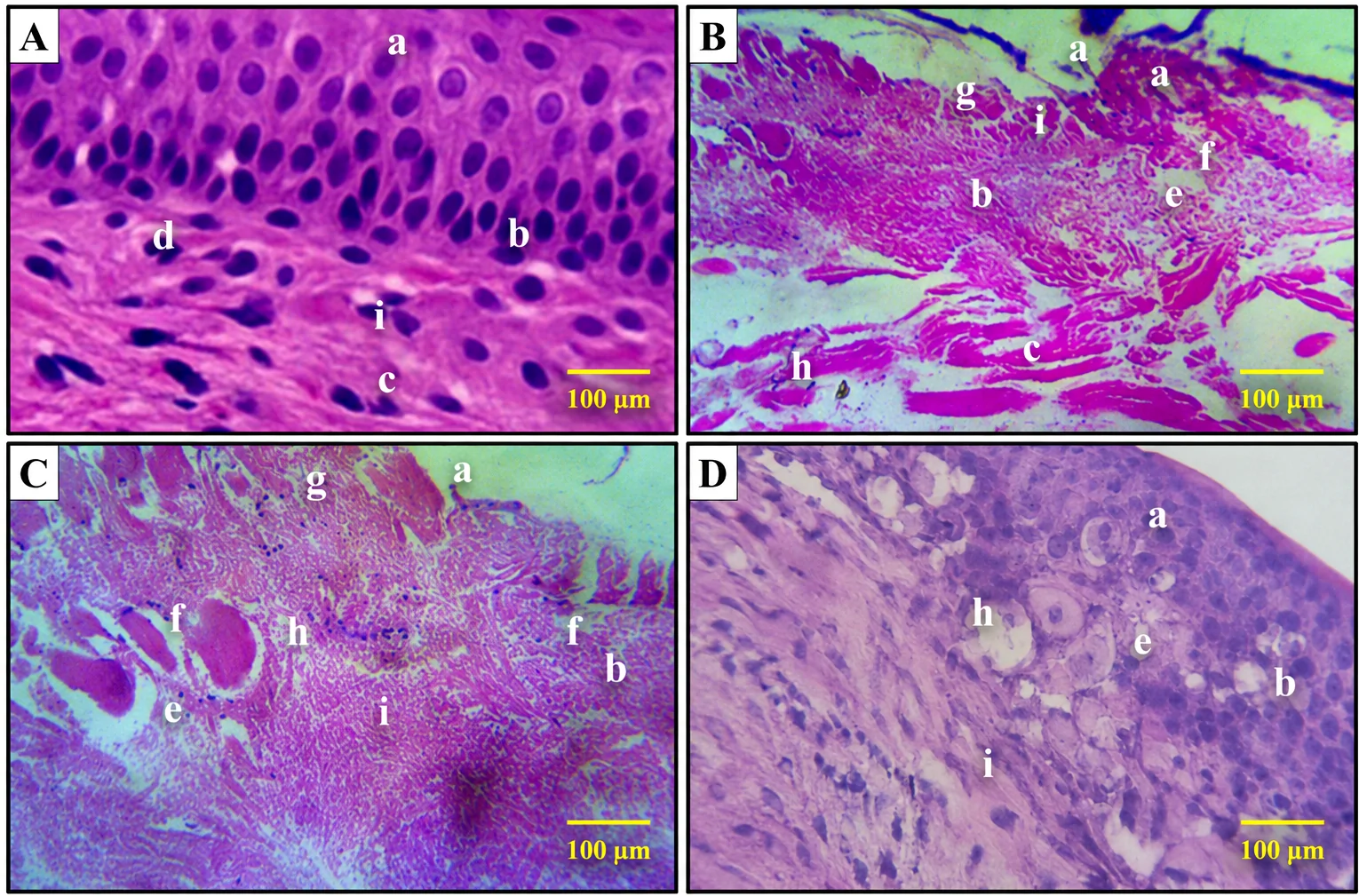
Histopathology of fish skin. **(A)** control fish, **(B, C)** diseased/dead fish **(D)** IgY treated fish. (a) Epidermis, (b) Dermis, (c) Scale, (d) Mucus cells, (e) Edema, (f) Necrosis, (g) Loss of architecture, (h) Inflammatory infiltrate, (i) Complete architecture, (j) Hemorrhage. H&E stain, 8µm thickness (No. of fish/tank=10, No. of trials 3). Magnification: ×400. Scale bar = 100 µm.

**Figure 8:**
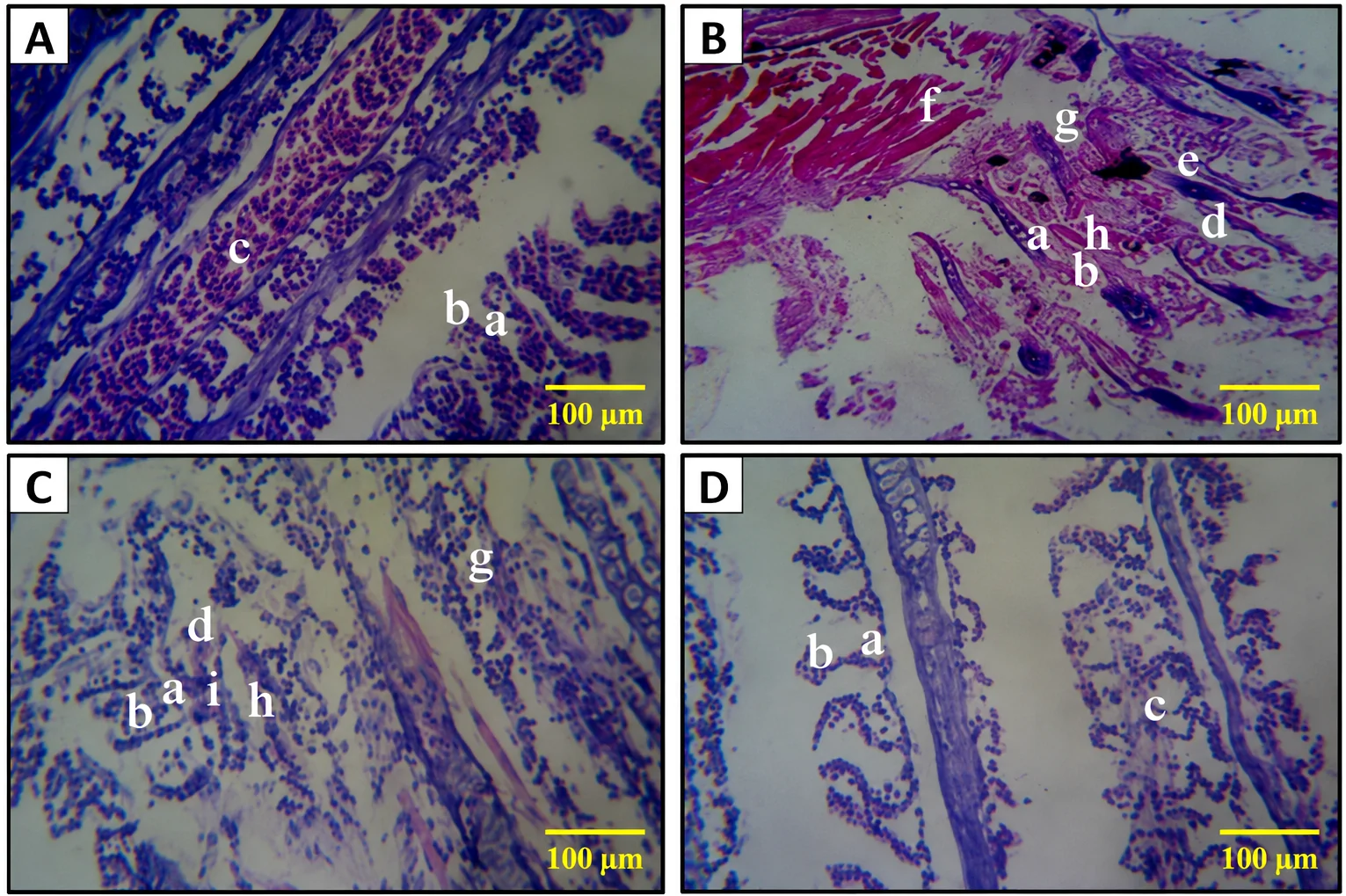
Histopathology of fish gills. **(A)** control fish, **(B, C)** diseased/dead fish **(D)** IgY treated fish. (a) Primary lamellae, (b) Secondary lamellae, (c) Blood vessels, (d) Edema, (e) Necrosis, (f) Hemorrhage, (g) Inflammatory infiltrate, (h) Disrupted epithelium, (i) Oedematous lamellae. H&E stain, 8µm thickness (No. of fish/tank=10, No. of trials 3). Magnification: ×400. Scale bar = 100 µm.

The fish from the control group had a normal, rigid structure of the gills (**Figure 8a**), while the infected gills exhibited necrosis, inflammation, and complete loss of architecture (**Figures 8b, c**). In contrast, the IgY-treated fish showed only mild symptoms of infection (**Figure 8d**).

**Figure 9:**
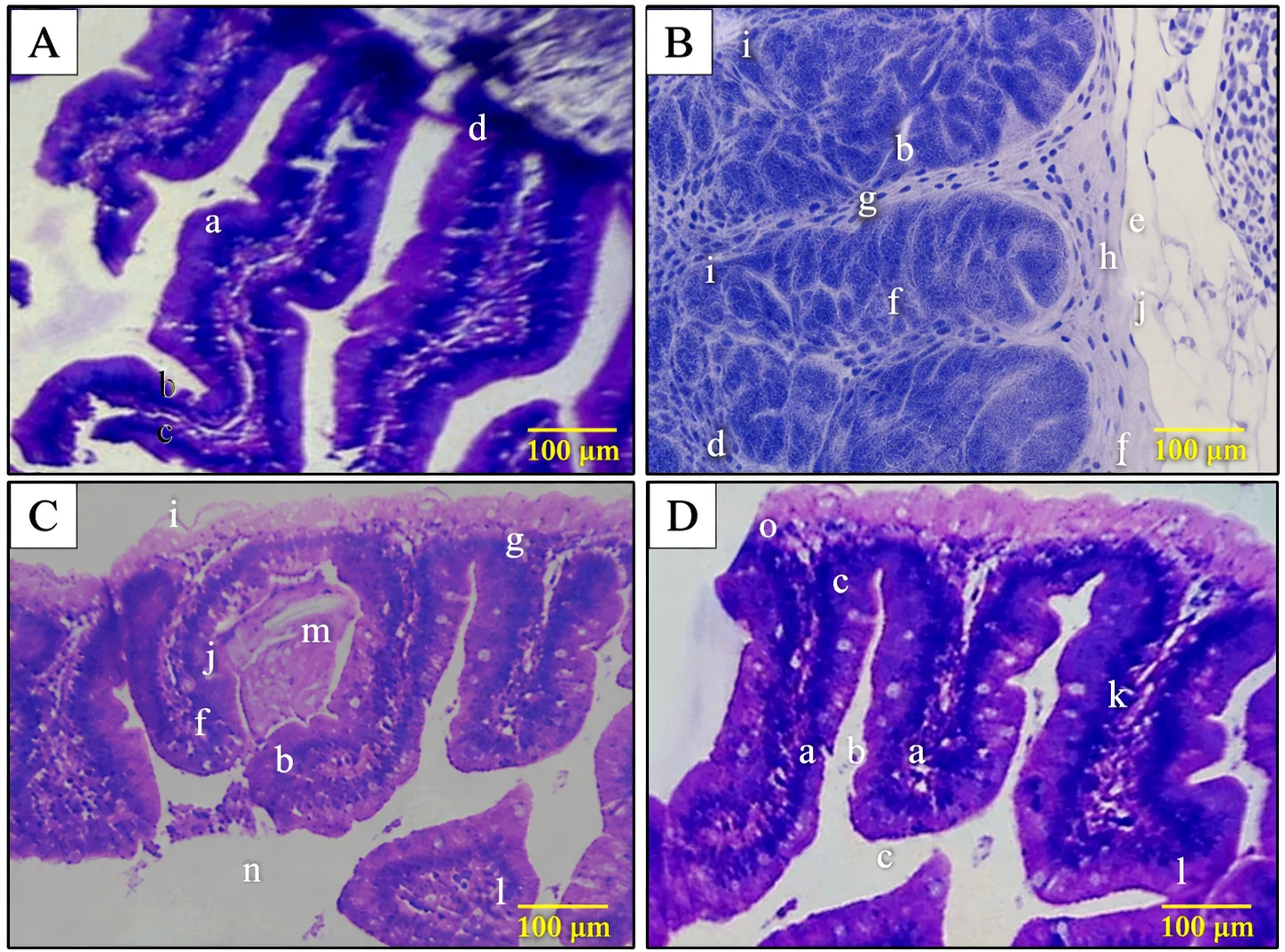
Histopathology of the fish small intestine. **(A)** control fish, **(B, C)** diseased/dead fish **(D)** IgY treated fish. The proximal small intestinal section shows: (a) Villi, (b) Epithelium, (c) Lamina propria, (d) Crypts (crypts of Lieberkühn), (e) Lumen, (f) Distorted, blunted villi, (g) Edema, (h) Inflammatory infiltrate, (i) Lamina propria expanded with inflammatory cells, (i) Disrupted epithelium, (k) Vacuole, (l) Necrosis, (m) Crypt hyperplasia, (n) Distorted intestinal crypts, (o) Inflammatory infiltrate. H&E stain, 8µm thickness (No. of fish/tank=10, No. of trials 3). Magnification: ×400. Scale bar = 100 µm.

The small intestine of fish from the control group showed normal well-structured tissues (**Figure 9a**), whereas the infected fish exhibited necrosis and gangrene in their small intestine (**Figures 9b, c**). The IgY-treated fish displayed a normal small intestine with the lamina propria showing mild inflammatory infiltrates (**Figure 9d**).

**Figure 10:**
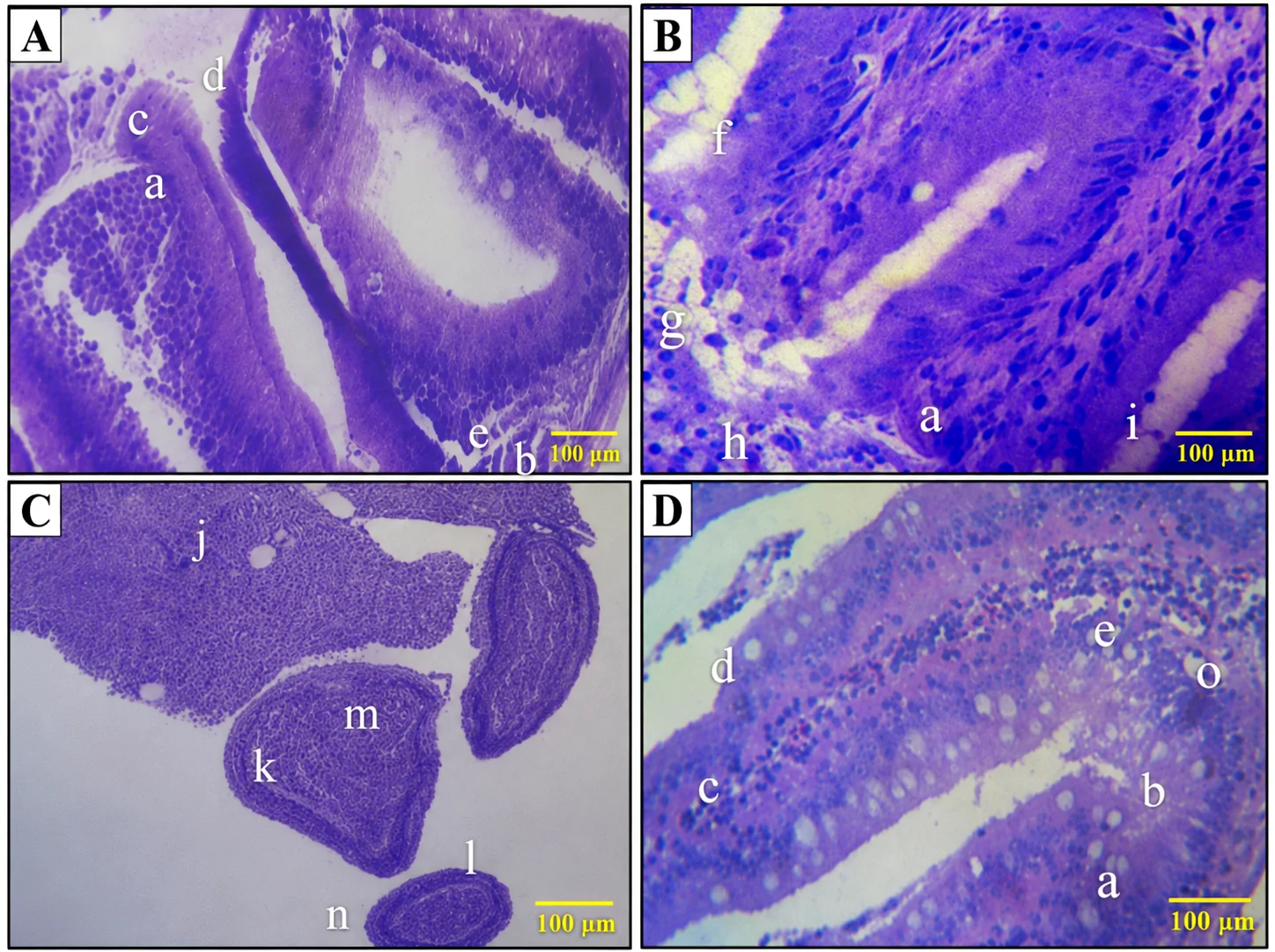
Histopathology of fish large intestine. **(A)** control fish, **(B, C)** diseased/dead fish **(D)** IgY treated fish. The distal large intestinal sections shows: (a) Crypts of Lieberkühn, (b) Mucosa, (c) Goblet cells/mucus, (d) Surface of epithelium, (e) Lamina propria, (f) ecrosis, (g) Aggregate, (h) Inflammation, (i) Gas gangrene, (j) Ulceration, (k) Inflammatory cell infiltration, (l) Vascular congestion, (m) Mucosal erosion, (n) Submucosal edema, (o) Scattered inflammatory infiltrate. H&E stain, 8µm thickness (No. of fish/tank=10, No. of trials 3). Magnification: ×400. Scale bar = 100 µm.

The untreated fish from the control group exhibited a fine structure of the large intestine (**Figure 10a**), whereas the pathogen-treated fish showed necrosis with inflammation and ulceration in the epithelial cells (**Figures 10b, c**). In contrast, the IgY-treated fish displayed only mild inflammatory infiltrates (**Figure 10d**).

## 4 Discussion

The opportunistic bacterium *A. hydrophila* remains one of the most significant threats to freshwater and marine aquaculture systems worldwide. Recent meta-analysis estimates a pooled prevalence of approximately 30.7% in aquatic food animals, with high rates of virulence gene carriage (≈ 71.2%) and widespread antimicrobial resistance (AMR), including resistance to penicillin (~80.7%), oxytetracycline (~69.9%) and macrolides (~67.8%) (26). The economic impact of such infections, through mortality, growth retardation, and increased production costs, is significant and escalating, especially in intensive aquaculture settings. Environmental stressors, such as elevated temperature, pH fluctuations, high ammonia levels, and low dissolved oxygen, further intensify *A. hydrophila* pathogenicity by weakening host immunity and promoting the expression of virulence genes (27).

In response, many producers depend heavily on broad-spectrum antibiotics (e.g., tetracyclines, sulfonamides). However, the repeated and often prophylactic use of antibiotics in aquaculture is strongly implicated in the rise of multidrug-resistant (MDR) and extensively drug-resistant (XDR) strains one study found 63.1% of isolates were MDR and 28.9% were XDR (28). Considering the zoonotic potential of *A. hydrophila*, antibiotic residues in fish, and horizontal gene transfer of AMR genes, the industry has a clear need to adopt alternative disease-control strategies.

In this context, immunoglobulin Y (IgY) technology provides a promising antibiotic-free option for disease control in aquaculture. IgY antibodies derived from the egg yolk of immunized hens can be produced in large quantities, carry minimal to no risk of promoting microbial resistance, and do not leave harmful residues in the environment or in fish tissues. Recent reviews emphasize IgY’s versatility across biomedical, environmental, and agricultural applications, including disease prevention in aquaculture through oral or feed-based delivery (29, 30). In recent aquaculture-specific trials, IgY antibodies targeting pathogen antigens have demonstrated effective passive immunization: for example, a recent study reported significant survival benefits and reduced tissue damage in fish challenged with *A. hydrophila* or *A. veronii* when fed IgY-supplemented diets (31).

The key advantages of the non-invasive antibody harvest method such as low allergenicity, ease of formulation into feed or sprays, and durability enhance its field applicability. Earlier purification techniques, like PEG–(NH_4_)_2_SO_4_ precipitation, remain efficient and cost-effective for IgY isolation (20). Many protocols enable hens to resume normal life after sampling, reinforcing the ethical and practical sustainability of IgY production (32). These attributes position IgY as a viable and environmentally responsible tool for disease management in aquaculture, aligning well with increasing regulatory and consumer pressure to reduce antibiotic use.

Despite the significant evolutionary divergence between fish and birds, fish species have been shown to mount vigorous immune responses when provided with heterologous antibodies such as immunoglobulin Y (IgY). Recent studies reveal that IgY can exhibit high affinity and avidity for aquatic pathogens and strong neutralizing capabilities (33). The mechanistic action of IgY in aquatic species has been characterised into several key modes of action including agglutination of pathogens, blockade of pathogen adherence to host tissues, opsonisation leading to enhanced phagocytosis, and neutralization of pathogen toxins or infective forms (34).

In our study, we first validated IgY neutralization *in vitro* using complementary assays, then proceeded to developing IgY-coated fish feed formulations. We found that passive vaccination *via* feed supplementation achieved an 80% survival rate when fish were fed a 3- 3.5% IgY-coated diet, which was markedly higher than the 60% survival observed with the curing method and 70% with the neutralization method alone.

This disparity highlights the critical ‘window of opportunity’ in passive immunization. In the prophylactic group, IgY was present in the intestinal lumen before pathogen exposure, allowing immediate binding to *A. hydrophila*. This likely reduced bacterial adhesion, agglutinated bacterial cells, and neutralized bacterial virulence factors, thereby limiting colonization and subsequent systemic dissemination. The lower efficacy observed in the therapeutic group suggests that once bacterial colonization and tissue invasion were established, orally administered IgY was less effective in controlling disease progression. The superior protection observed in the prophylactic group is likely attributable to the presence of pathogen-specific IgY at the site of pathogen entry before infection, thereby reducing bacterial colonization and the severity of infection. Similar immunomodulatory effects following oral IgY administration have been reported in fish, crab, shrimp and other aquatic species, where passive antibodies improved immune parameters, survival and resistance to bacterial infections (33, 35–37).

These outcomes align with the emerging consensus that oral delivery of IgY antibodies is a potent strategy for passive immunisation in both terrestrial and aquatic animals (38). For example, prior research in marine aquaculture using IgY- Encapsulated feed in small abalone challenged with *Vibrio alginolyticus* produced survival rates of approximately 65-70% (39). An additional advantage of using avian IgY in fish is that these antibodies do not trigger Fc- mediated complement activation in fish, thereby reducing the risk of unwanted immune side- effects. Indeed, in several studies, IgY supplementation has been linked to improved haematological and immune parameters in fish, indicating a general enhancement of health status beyond simple pathogen neutralization (40).

Histopathology studies on various fish tissues were conducted as a continuation of this work, comparing test findings to the control. In our study, fish fed with IgY showed minimal loss of tissue structure, such as skin, gills, and both small and large intestines. We hypothesize that the oral administration of IgY-coated fish feed significantly reduced *A. hydrophila*-induced septicaemia in *C. idella*. Specifically, this treatment will preserve internal tissue integrity, reduce systemic histopathological damage, and enhance the overall survival rate compared to non-immunized controls. An assessment of the protective effects of IgY against *Spiroplasma eriocheiris* infection in Chinese mitten crabs involved histopathological examination of tissues from crabs fed IgY-infused feed or given intraperitoneal injections of IgY, compared to crabs that did not receive any IgY (41). The results indicate a potential protective role of IgY against this infection, consistent with our findings.

Beyond its therapeutic efficacy, the findings of the present study indicate that IgY-coated fish feed has the potential to support more sustainable disease management practices in aquaculture. The reduced reliance on antibiotics demonstrated in this study is consistent with the objectives of Sustainable Development Goal (SDG) 14 (Life Below Water) by promoting environmentally responsible aquaculture practices and may indirectly contribute to SDG 2 (Zero Hunger) through improved fish health and productivity. The use of IgY as an alternative to antibiotics may also contribute to efforts aimed at reducing antimicrobial resistance and minimizing antibiotic residues in aquaculture products. In the present study, prophylactic administration of IgY-coated feed improved fish survival and reduced histopathological damage, suggesting that IgY could serve as a promising immunotherapeutic approach for disease prevention under experimental conditions. However, further large-scale field trials and economic evaluations are required before its commercial application in aquaculture can be recommended.

## 5 Conclusion

The present study demonstrates that IgY antibody-coated fish feed provides an effective and sustainable alternative to antibiotics for preventing and controlling *A. hydrophila* infections in *C. idella*. Oral administration of IgY significantly improved fish survival rates and decreased histopathological lesions in vital tissues such as the gills, skin, and intestines, indicating strong protective and immunomodulatory effects. The high efficacy of IgY in curing, neutralizing, and vaccinating highlights its potential as a safe, eco-friendly immunotherapeutic agent for the management of aquaculture diseases. By reducing dependence on antibiotics, IgY technology has the potential to mitigate antimicrobial resistance and minimize environmental contamination, thereby contributing to more sustainable aquaculture practices. Nevertheless, further validation under commercial farming conditions is warranted before large-scale implementation.

## Data Availability

The raw data supporting the conclusions of this article will be made available by the authors, without undue reservation.
